# Supplementary material for: Intermolecular β‑sheet Formation Guides the Interaction between Ubiquitin-like Modifier FAT10 and Adapter Protein NUB1L
Source: J Am Chem Soc. 2026 May 20;148(21):22219–31. doi: 10.1021/jacs.6c05291 (PMC13244463; doi:10.1021/jacs.6c05291)
Supplement: Supplementary file 1 [file ja6c05291_si_001.pdf]

Supporting Information

Intermolecular β-sheet formation guides the interaction between ubiquitin-like modifier FAT10 and adapter protein NUB1L

Charlotte Weiss[1], Sarah Overall[2], Nicola Catone[3], Alexander B. Barnes[2], Annette Aichem[3], and Guinevere Mathies[1,*]

[1]Department of Chemistry, University of Konstanz, 78464 Konstanz, Germany.

[2]Institute of Molecular Physical Science, ETH Zürich, 8093 Zürich, Switzerland.

[3]Institute of Cell Biology and Immunology Thurgau, 8280 Kreuzlingen, Switzerland.

Contents

1. Protein expression and purification 2
2. Packing of samples for MAS NMR 3
3. MAS NMR spectroscopy 5
4. ^{1}H-^{15}N cross-polarization spectra 8
5. ^{13}C-^{13}C and ^{15}N-^{13}C spectra of microcrystalline N-FAT10-C0 9
6. Chemical shifts of microcrystalline N-FAT10-C0 10
7. Torsion angle analysis of microcrystalline N-FAT10-C0 12
8. ^{13}C-^{13}C and ^{15}N-^{13}C spectra lyophilized/rehydrated N-FAT10-C0 13
9. Chemical shifts of lyophilized/rehydrated N-FAT10-C0 14
10. Torsion angle analysis of lyophilized/rehydrated N-FAT10-C0 16
11. ^{13}C-^{13}C and ^{15}N-^{13}C spectra of N-FAT10-C0 in complex with NUB1L 17
12. Temperature dependence of N-FAT10-C0 in complex with NUB1L 18
13. Chemical shifts of N-FAT10-C0 in complex with NUB1L 19
14. Torsion angle analysis of N-FAT10-C0 in complex with NUB1L 20
15. AlphaFold-Multimer prediction for the interaction of N-FAT10-C0 and NUB1L 21
16. Double-REDOR experiments 22

*E-mail: guinevere.mathies@uni-konstanz.de

1. Protein expression and purification

U-^{13}C,^{15}N-N-FAT10-C0

Cysteine-free N-domain of human FAT10 (amino acids 5-86; C7T, C9T) was expressed as a His_6-GST-fusion protein in *E. coli* BL21-CodonPlus(DE3)-RIPL competent cells (Agilent Technologies). The pETM-30 plasmid was kindly provided by the Institute of Cell Biology and Immunology Thurgau (Kreuzlingen, Switzerland); a TEV protease cleavage site C-terminal to the purification tag results in an additional glycine residue at the N-terminus after cleavage. For uniform ^{13}C and ^{15}N labelling, 3.6 g of U-$^{13}C_6$-D-glucose and 0.5 g of ^{15}N-ammonium chloride per litre of M9 minimal medium were added as exclusive sources of carbon and nitrogen. Bacteria cells were grown at 37 °C to an OD_{600} of 0.5-0.6, induced with 0.4 mM IPTG at 21 °C overnight, and harvested by centrifugation (RCF 4000 g, 10 min, 8 °C). Harvested cells were lysed in lysis buffer (20 mM TRIS-HCl (pH 8.0), 300 mM NaCl, 10 mM imidazole, 10 % v/v glycerol, 0.1 % v/v Triton X-100, 100 µg/mL lysozyme, 1 mM PMSF and EDTA-free protease inhibitor). After sonication, cell debris was removed by centrifugation (RCF 47000 g, 30 min, 8 °C). The supernatant was filtered and applied to Ni-NTA Agarose beads (Macherey-Nagel) for affinity chromatography. The protein was eluted with elution buffer (20 mM TRIS-HCl (pH 8.0), 300 mM NaCl and 500 mM imidazole) and buffer exchanged to binding buffer (20 mM TRIS-HCl (pH 8.0), 300 mM NaCl and 10 mM imidazole). His-tagged TEV protease was allowed to act overnight (5 µg of protease per 1 mg of recombinant protein). After cleavage, His-tagged TEV protease and cleavage byproducts were separated by a second Ni-affinity chromatography using binding buffer. The volume of the flow-through containing the U-^{13}C,^{15}N-N-FAT10-C0 was reduced to 5 mL and filtered. Further purification was achieved by size-exclusion chromatography (Cytiva, formerly Amersham Biosciences, ÄKTA pure chromatography system equipped with a HiLoad 16/600 Superdex 75 pg column) yielding up to 7 mg per litre of M9 medium. Before preparation of the samples for MAS NMR, U-^{13}C,^{15}N-N-FAT10-C0 was in 20 mM HEPES (pH 7.5) and 150 mM NaCl and concentrated to specified values (Amicon Ultra-15 centrifugal filter unit, MWCO 3 kDa).

NUB1L

Human NUB1L (amino acids 2-615) was expressed as a His_6-SUMO-fusion protein also in *E. coli* BL21-CodonPlus(DE3)-RIPL competent cells. The pSUMO plasmid was kindly provided by the Institute of Cell Biology and Immunology Thurgau (Kreuzlingen, Switzerland). Bacteria cells were grown in LB medium at 37 °C to an OD_{600} of 0.7, induced with 0.4 mM IPTG at 21 °C overnight and harvested by centrifugation (RCF 4000 g, 10 min, 8 °C). The harvested cells were lysed in lysis buffer (20 mM TRIS-HCl (pH 7.5), 150 mM NaCl, 20 mM imidazole, 1 mM TCEP, 10 % v/v glycerol, 0.1 % v/v Triton X-100, 100 µg/mL lysozyme, 1 mM PMSF and EDTA-free protease inhibitor). After sonication, cell debris was removed by centrifugation (RCF 27000 g, 30 min, 8 °C). The supernatant was filtered and applied to Ni-NTA Agarose beads for affinity chromatography. The protein was eluted with elution buffer (20 mM TRIS-HCl (pH 7.5), 150 mM NaCl, 500 mM imidazole and 1 mM TCEP) and buffer exchanged to binding buffer (20 mM TRIS-HCl (pH 7.5), 150 mM NaCl, 20 mM imidazole and 1 mM TCEP). His-tagged Ulp1 protease was added overnight (4 µg of protease per 1 mg of recombinant protein). After cleavage, His-tagged Ulp1 protease and cleavage byproducts were separated by a second Ni-affinity

chromatography using binding buffer. The volume of the flow-through containing (natural abundance) NUB1L was reduced to 5 mL and filtered. Further purification was achieved by size-exclusion chromatography yielding up to 15 mg of pure NUB1L per litre of LB medium. To form the complex with N-FAT10-C0, the protein was in 20 mM HEPES (pH 7.5), 150 mM NaCl, and 1 mM TCEP, concentrated to approximately 20 mg/mL (Amicon Ultra-15 centrifugal filter unit, MWCO 30 kDa).

2. Packing of samples for MAS NMR

Microcrystalline N-FAT10-C0

Microcrystals of U-^{13}C,^{15}N-N-FAT10-C0 were grown following an adapted version of the crystallization protocol of Aichem *et al.*[1], with the sitting-drop vapor diffusion method. Equal volumes of protein at a concentration of approximately 20 mg/mL and crystallization solution (3.2 M $(NH_4)_2SO_4$ and 0.1 M citric acid (pH 2.5)) were mixed in drops of 25 µL. Each drop was equilibrated against 0.75 mL of crystallization solution supplemented with additional 150 mM NaCl. After keeping the crystallization trays at 4 °C for four days, microcrystals were harvested. Ultracentrifugation was used to assure dense packing in a 3.2 mm MAS rotor. A home-made packing tool[2] containing the protein suspension and MAS rotor was centrifuged for 2 h at RCF 210000 g and 4 °C in a Beckman Coulter Optima L90K ultracentrifuge. The supernatant was removed and packing efficiency was assessed by UV-Vis absorbance measurements of the supernatant at 280 nm. The rotor, filled with an estimated 13.5 mg of microcrystalline U-^{13}C,^{15}N-N-FAT10-C0, was closed with a Vespel cap.

Lyophilized/rehydrated N-FAT10-C0

After concentration to approximately 8 mg/mL, U-^{13}C,^{15}N-N-FAT10-C0 was lyophilized, ground, and packed into a 3.2 mm MAS rotor. The rotor containing an estimated 14.5 mg of protein was closed with a Vespel cap. Rehydration was accomplished by adding Milli-Q water in amounts of 1 µL while monitoring the ^{1}H-^{13}C cross-polarization spectrum. After addition of a total of 7 µL, no further improvement in spectral resolution was observed.

N-FAT10-C0 in complex with NUB1L

Complex formation of N-FAT10-C0 and NUB1L was verified with size-exclusion chromatography (Figure S1). To prepare the complex for MAS NMR spectroscopy, co-sedimentation into the rotor was used. 1 mL of U-^{13}C,^{15}N-N-FAT10-C0 at a concentration of 3 mg/mL was mixed with 1 mL of natural abundance NUB1L with a concentration of 20 mg/mL, leading to a molar ratio of 1:1. The packing tool containing this solution was centrifuged at RCF 210000 g and 4 °C. As the holding capacity of the packing tool is only 1 mL, the run was, over the course of several days, repeatedly interrupted to remove supernatant and add the original mixture in full. Packing efficiency was assessed by UV-Vis absorbance measurements of the supernatant at 280 nm. The 3.2 mm rotor was closed with a Vespel cap.

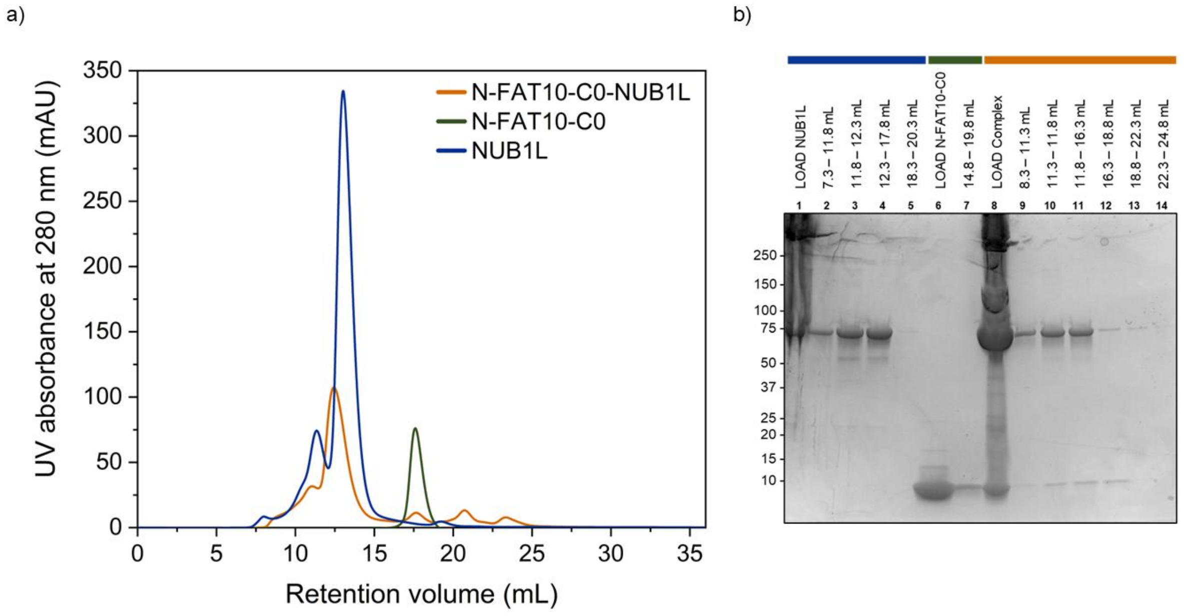

Figure S1: *Size-exclusion chromatography, on a Cytiva ÄKTA pure chromatography system equipped with a Superdex 200 Increase 10/300 GL column, and subsequent SDS-PAGE analysis. (a) Individual runs of isolated N-FAT10-C0 (green) and isolated NUB1L (dark blue) are shown together with a mixture of both N-FAT10-C0 and NUB1L (orange). Molar amounts were adjusted to be the same in each run. The shift to smaller retention volumes in the orange chromatogram indicates a larger molecular weight and thereby complex formation. (b) SDS-PAGE analysis before and after size-exclusion chromatography. 5 µL of gel sample buffer with SDS (reducing conditions) was added to 20 µL of each sample (loads for size-exclusion chromatography as well as collected fractions containing protein). After boiling for 5 min at 95 °C, samples were loaded on a gradient gel and separated in 30 min by applying a constant voltage of 200 V, followed by colloidal Coomassie staining. For molecular weight estimation, Precision Plus Protein Dual Color Standards (Bio-Rad) were used.*

3. MAS NMR spectroscopy

MAS NMR experiments were performed at 18.8 T (^{1}H Larmor frequency of 800.3 MHz) in the NMR Core Facility at the University of Konstanz and at 20.0 T (^{1}H Larmor frequency of 850.2 MHz) at ETH Zürich. The console was Bruker Avance NEO or Avance III. Bruker 3.2 mm E-free HCN MAS probes were used, together with regular-wall 3.2 mm ZrO rotors (maximum sample volume 32.1 µL). During experiments, the sample was kept at a temperature of approximately 4 °C, unless noted otherwise. Calibration of the Bruker Cooling Unit (BCU) II was performed by monitoring the chemical shift of ^{79}Br in KBr powder.[3] The carbon dimension was referenced indirectly to DSS in D_2O (0.5 % by weight),[4] i.e., the ^{13}C adamantane methylene peak is observed at 40.49 ppm. The nitrogen dimension was referenced to liquid ammonia at 25 °C.[5] Table S1 provides an overview of the experiments performed per sample.

Table S1: *MAS NMR spectra obtained for each sample.*

Experiment	Field (T)	Number of scans	^{15}N-^{13}C /^{13}C-^{13}C mixing (ms)	Temperature (°C)	MAS frequency (kHz)	Measurement time
**Microcrystalline U-^{13}C,^{15}N-N-FAT10-C0**						
hC	18.8	256		4	14.5	
hN	18.8	128		4	14.5	
DARR	18.8	32	10	4	14.5	16 h
ZF TEDOR	18.8	32	0.52	4	14.5	7 h
ZF TEDOR DARR	18.8	4	1.1/40	4	14.5	7 d 21 h
**Lyophilized/rehydrated U-^{13}C,^{15}N-N-FAT10-C0**						
hC	18.8	256		4	14.5	
hN	18.8	512		4	19.0	
dREDOR CCP	20.0	1024		4	19.0	
dREDOR NCP	20.0	512		4	19.0	
DARR	18.8	32	10	4	14.5	22 h
ZF TEDOR	18.8	128	0.84	4	19.0	15 h
ZF TEDOR DARR	18.8	8	0.84/40	4	19.0	5 d 22 h
NCOCX	20.0	32 (2x)	55.6 (CORD)	4	19.0	3 d (2x)
**U-^{13}C,^{15}N-N-FAT10-C0 in complex with natural abundance NUB1L**						
hC	20.0	128/128/256		-10/-2/4	19.0	
hN	20.0	2048		4	19.0	
C	20.0	128		-10/-2/4	19.0	
dREDOR CCP	20.0	2048		4	19.0	
dREDOR NCP	20.0	2048		4	19.0	
DARR	20.0	256	10	4	19.0	3 d
dREDOR DARR	20.0	1536	20	4	19.0	5 d 18 h
ZF TEDOR	20.0	256 (2x)	1.26	4	19.0	1 d 12 h (2x)
ZF TEDOR DARR	20.0	8 (3x)	1.26/50	4	19.0	5 d 5 h (3x)
NCOCX	20.0	64	55.6 (CORD)	-2	19.0	6 d

Typical π/2-pulse lengths were 3.0-3.5 µs for ^{1}H, 3.0-5.0 µs for ^{13}C and 6.0-10.5 µs for ^{15}N. ^{1}H-^{13}C and ^{1}H-^{15}N cross-polarization (CP)[6] are realized using a linearly ramped RF field on the ^{1}H channel (either from 70 or 90 to 100 % amplitude); the centre of the ramp was set to match the n = +1 Hartmann-Hahn condition.[7,8] Swept-frequency two-pulse phase modulation (SW_f-TPPM)[9] or small phase incremental alternation with 64 steps (SPINAL-64)[10] were applied for proton decoupling. The States-TPPI method[11] was used for phase-sensitive detection in the indirect dimensions.

The ZF TEDOR DARR sequence provides NCACX and NCOCX backbone connections within one three-dimensional spectrum, but at the cost of large spectral widths. To keep the number of increments in the indirect dimensions under control, it sufficed for microcrystalline N-FAT10-C0 to truncate the free induction decay along the indirect ^{13}C dimension and pad it using linear forward prediction. For lyophilized/rehydrated N-FAT10-C0 and the N-FAT10-C0/NUB1L complex, we used aliasing. At a spinning frequency of 19.0 kHz, a convenient strategy is to set the dwell time of the indirect ^{13}C dimension equal to one rotor period and the carrier frequency to approximately 50 ppm.[12] The carbonyl resonances are now observed at the positions of their spinning sidebands just above the aliphatic region.

Before running a ^{15}N-^{13}C-^{13}C ZF TEDOR DARR experiment, a ^{15}N-^{13}C ZF TEDOR experiment served to set the acquisition parameters correctly, including the mixing time and the duration of the REDOR π-pulses. The z-filter delays were set to ~200 µs, matching a multiple of the rotor period. Table S2 gives a complete overview of the acquisition parameters per experiment and sample.

For free induction decays with unnecessary long acquisition times, the number of effective points was adjusted accordingly and zero-filling was applied. 1D spectra were baseline corrected, but no apodization was used. For 2D and 3D spectra, 45°- or 72°-shifted squared-sine bell apodization (corresponding to an SSB value of 4 or 2.5 in TopSpin and an offset of 0.25 or 0.4 in NMRPipe) was used. The resolution of the ZF TEDOR DARR spectrum of microcrystalline N-FAT10-C0 and of the dREDOR DARR spectrum of N-FAT10-C0/NUB1L was improved by forward linear prediction to 1.5 and, respectively, 2.0 times the number of the original data points in the indirect carbon dimension.

Table S2: *Acquisition parameters for MAS NMR experiments.*

Experiment	CP contact time (ms)		Radio frequency field strength (kHz)								Dwell time (μs)			Acquisition time (ms)			Carrier position (ppm)			Recycle delay (s)
	^{1}H-^{13}C/ ^{1}H-^{15}N CP	^{15}N-^{13}C DCP	^{1}H-^{13}C/^{1}H-^{15}N CP		^{15}N-^{13}C DCP		π/2- and π-pulses			Decoupling										
			$^{1}H^{\#}$	^{13}C/^{15}N	^{15}N	$^{13}C^{\#}$	^{1}H	^{13}C	^{15}N	^{1}H	^{13}C	^{13}C	^{15}N	^{13}C	^{13}C	^{15}N	^{13}C	^{13}C	^{15}N	
**Microcrystalline U-^{13}C,^{15}N-N-FAT10-C0**																				
hC	0.9		67.9^{r}	50.0			83.3			71.4, SW_f-TPPM	8.5			17.4			100			4.0
hN	1.0		74.8^{r}	50.0			71.4			71.4, SPINAL-64			12.0			12.3			104	4.0
DARR	1.0		75.9^{r}	50.0			83.3	50.0		71.4, SPINAL-64	11.3	23.0		23.1	6.9		100	100		3.0
ZF TEDOR	0.9		67.9^{r}	50.0			83.3	50.0	23.8	71.4, SW_f-TPPM	12.0		69.0	13.2		10.3	100		104	2.5
ZF TEDOR DARR	0.9		67.9^{r}	50.0			83.3	50.0	23.8	71.4, SW_f-TPPM/ SPINAL-64	11.3	23.0	69.0	13.6	2.9	8.8	100	100	104	2.5
**Lyophilized/rehydrated U-^{13}C,^{15}N-N-FAT10-C0**																				
hC	0.9		67.9^{r}	50.0			83.3			71.4, SPINAL-64	6.1			25.0			100			3.0
hN	0.7		64.3^{r}	41.7			83.3			71.4, SPINAL-64			10.0			20.5			104	3.0
dREDOR CCP	4.0		63.0^{r}	38.7			100	50.0	48.1	98.3, SPINAL-64	5.8			11.9			100			2.0
dREDOR NCP	4.0		70.6^{t}	46.5			100	50.0	48.1	98.3, SPINAL-64			14.6			29.9			122	2.0
DARR	0.9		67.9^{r}	50.0			83.3	50.0		71.4, SPINAL-64	11.3	23.0		23.1	9.3		100	100		3.0
ZF TEDOR	0.8		72.2^{r}	50.0			83.3	83.3	36.2	90.6/83.3, SW_f-TPPM	12.0		52.6	24.6		5.3	100		87	2.0
ZF TEDOR DARR	0.9		72.2^{r}	50.0			83.3	83.3	36.2	90.6/83.3, SW_f-TPPM; 83.3, SPINAL-64	6.1	52.6	105.3	12.5	5.0	8.4	50	50	87	2.0
NCOCX	0.7	1.3	79.5^{t}	41.7	24.0	56.2^{t}	83.3	62.5	41.7	90.0, SW_f-TPPM; 100 kHz, CW	11.0	210.5	210.5	22.5	5.1	8.4	85	85†	122	2.0
**U-^{13}C,^{15}N-N-FAT10-C0 in complex with natural abundance NUB1L**																				
hC	1.0		72.7^{r}	50.0			83.3			83.3, SPINAL-64/ SW_f-TPPM*	5.8			11.9			100			3.0
hN	0.8		75.6^{t}	48.3			100			98.2, SPINAL-64			14.6			29.9			122	2.0
C								62.5		83.3, SW_f-TPPM	5.8			11.9			100			4.0
dREDOR CCP	4.0		69.1^{r}	40.8			100	50	47.2	98.2, SPINAL-64	5.8			11.9			100			2.0
dREDOR NCP	4.0		75.6^{t}	48.3			100	50	47.2	98.2, SPINAL-64			14.6			29.9			122	2.0
DARR	1.0		72.7^{r}	50.0			83.3	50.0		83.3, SPINAL-64	11.0	21.1		11.3	5.2		100	100		2.0
dREDOR DARR	4.0		69.1^{r}	40.8			100	50	47.2	98.2, SPINAL-64	11.0	21.1		22.5	1.6		100	100		2.0
ZF TEDOR	1.0		72.7^{r}	50.0			83.3	62.5	38.5	96.2/83.3, SW_f-TPPM	11.0		52.6	11.3		6.5	100		104	2.0
ZF TEDOR DARR	1.0		72.7^{r}	50.0			83.3	62.5	38.5	96.2/83.3, SW_f-TPPM; 83.3, SPINAL-64	6.1	52.6	105.3	12.5	5.0	7.4	56	56	83	2.0
NCOCX	0.8	1.0	80.5^{t}	50.0	11.0	9.4^{t}	83.3	50.0	41.7	90.0, SW_f-TPPM; 90 kHz, CW	11.0	210.5	210.5	22.5	5.1	8.4	85	85†	122	2.0

$^{\#}$Corresponds to 100 % of the amplitude. ^{r}Linear ramp. ^{t}Tangent. *Decoupling sequence for ^{1}H-^{13}C CP temperature series. †For ^{15}N-^{13}C SPECIFIC-CP, the CO carrier was centred at 174 ppm.

4. ^{1}H-^{15}N cross-polarization spectra

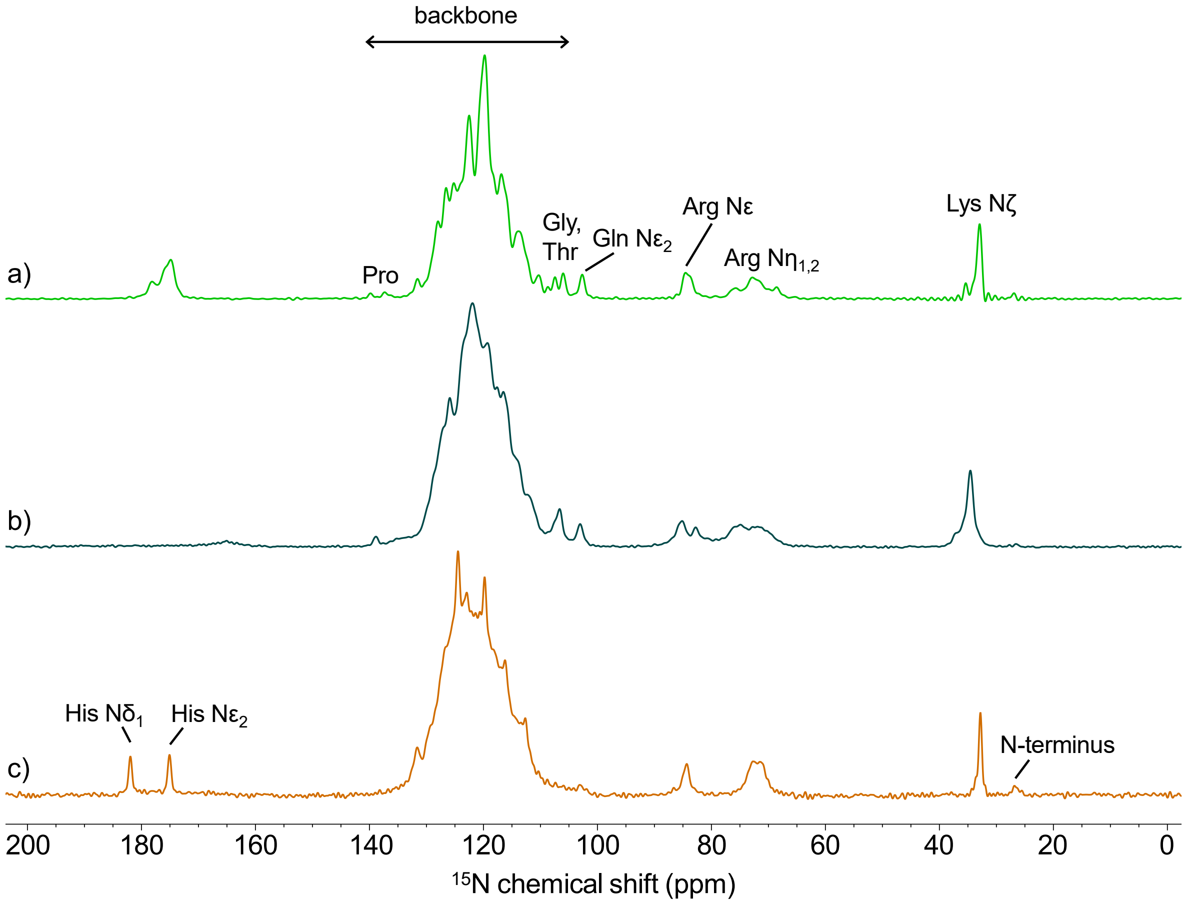

Figure S2: *^{1}H-^{15}N cross-polarization spectra of (a) microcrystalline N-FAT10-C0, (b) lyophilized/rehydrated N-FAT10-C0, and (c) N-FAT10-C0 in complex with NUB1L.*

5. ^{13}C-^{13}C and ^{15}N-^{13}C spectra of microcrystalline N-FAT10-C0

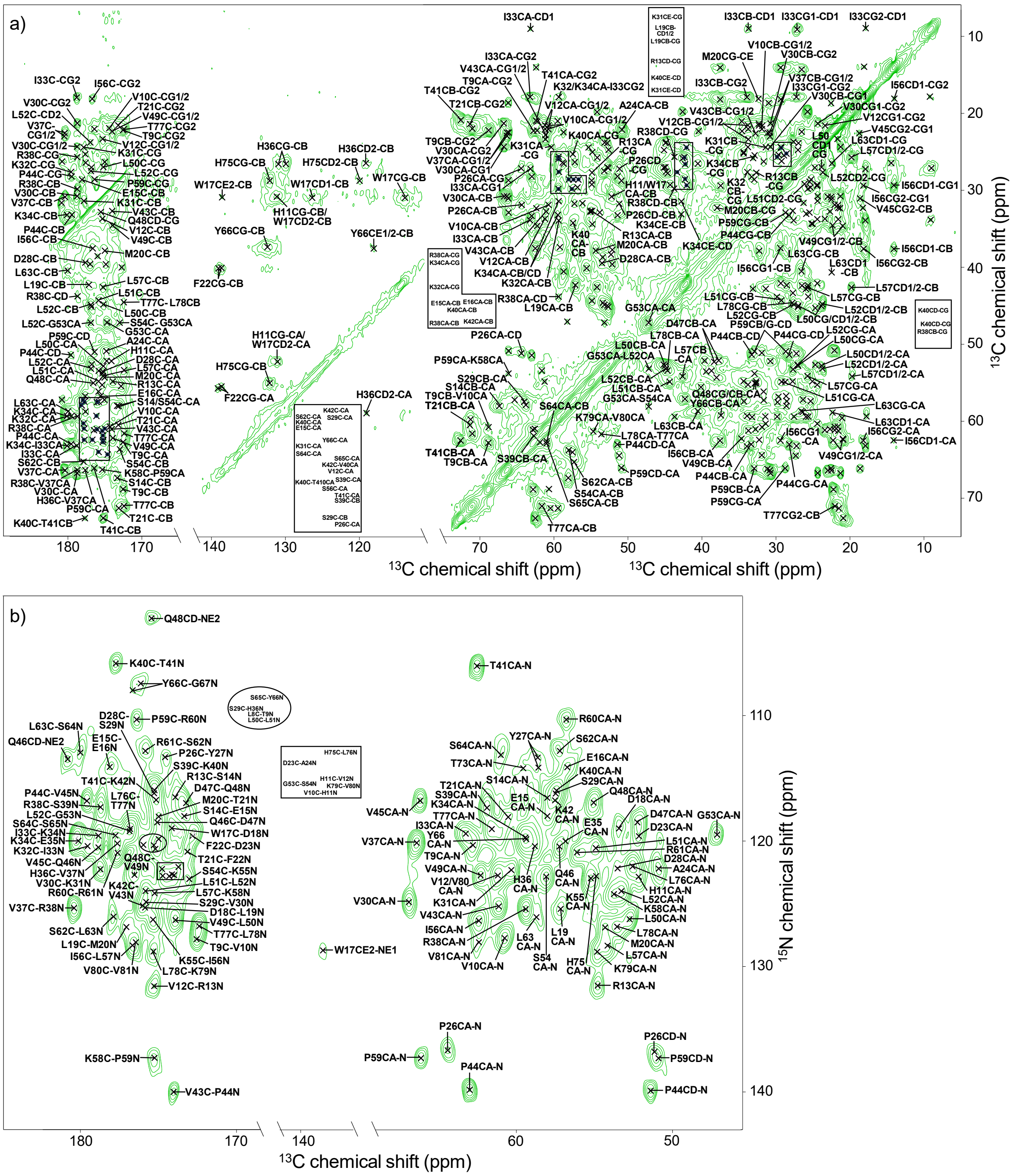

Figure S3: *(a) ^{13}C-^{13}C DARR spectrum of microcrystalline N-FAT10-C0. The spectrum is dominated by one- and two-bond transfers, but some cross peaks from three-bond transfers are discernible from the noise, e.g. between C' and $C_{\gamma 2}$, C_{α} and $C_{\delta 1}$, and $C_{\gamma 2}$ and $C_{\delta 1}$ of I33 and I53. Some inter-residue contacts are visible, e.g. T9C_{β}-V10C_{α} and G53C_{α}-S54C_{α}. To maintain readability, only labels are included of residues that are also observed in the ^{13}C-^{13}C DARR spectrum of lyophilized/rehydrated N-FAT10-C0, i.e., cross peaks from D18, F22, D23, H36, V45, Q46, K55, K58, R60, R61, T73, H75, L76, K79, V80 and V81 are not labeled in the carbonyl and aliphatic regions. (b) ^{15}N-^{13}C ZF TEDOR spectrum of microcrystalline N-FAT10-C0.*

6. Chemical shifts of microcrystalline N-FAT10-C0

Table S3: *^{15}N and ^{13}C chemical shifts (ppm) of microcrystalline U-^{13}C,^{15}N-N-FAT10-C0.*

Residue	N	C'	Cα	Cβ	Cγ	Cδ	Other
L8[a]		175.3	54.9	44.3	26.1	22.2, 21.1	
T9	120.3	172.6	62.8	68.8	22.2		
V10	127.8	174.3	60.7	31.9	22.0, 21.6		
H11	122.8	174.3	52.2	30.8	131.1	119.0	
V12	122.6	175.3	61.2	33.7	23.4, 21.6		
R13	131.5	173.9	54.8	32.7	27.7	43.3	Nε: 84.7
S14	116.5	173.4	58.0	67.4			
E15	118.0	178.1	58.0	28.6	33.4		
E16	114.1	175.6	56.7	28.6	33.7		
W17		174.1	52.2	30.8	114.0	126.4, 131.2	Cε2: 138.6, Cη2: 125.1, Cζ2: 114.4, Nε1: 128.7
D18	119.0	175.9	53.4	38.7			
L19	125.3	177.1	57.1	42.3	26.6	25.7	
M20	126.9	175.0	54.3	37.9	32.3		Cε: 18.2
T21	118.0	173.1	60.5	71.3	21.5		
F22[m]	120.8	172.8	55.5	40.1	139.1		
D23	119.6	174.8	52.2	39.6	177.0		
A24	122.3	174.7	50.9	22.1			
N25	120.7	175.0	51.2	44.3			
P26	136.7	174.6	64.4	31.8	27.2	51.2	
Y27[m]	113.3	174.8	58.6	36.9			Cζ: 156.8
D28[m]	122.2	175.3	53.5	39.5	177.6		
S29	116.2	175.8	57.5	64.0			
V30	124.8	178.7	66.8	30.8	24.4, 21.3		
K31	122.2	177.9	60.3	31.4	24.4	29.4	Cε: 42.1, Nζ: 33.0
K32[a]	120.1	179.6	59.3	33.9	27.6		
I33	119.6	178.8	63.2	33.9	27.2, 17.9	9.1	
K34[a]	119.5	180.1	59.4	33.1	25.9	31.4	Cε: 42.8, Nζ: 35.1
E35	120.0	175.8	56.9	28.4			
H36[m]	120.3	177.6	58.8	26.4	129.9	119.1	Cε1: 138.2, Nδ1:175.8, Nε2: 174.4
V37	120.2	180.4	66.4	30.8	23.0, 22.5		
R38	125.4	178.7	59.4	29.8	25.7	43.8	Cζ: 159.3, Nε: 83.7, Nη1: 75.8, Nη2: 68.7
S39	117.3	175.3	61.9	62.7			
K40	116.0	177.8	57.4	34.3	25.3	28.6	Cε: 42.3, Nζ: 33.1
T41	106.0	175.2	62.4	72.6	20.9		
K42	116.7	176.1	57.6	29.8			
V43	125.3	174.0	61.1	32.8	22.8		
P44	139.8	179.5	63.0	33.3	28.2	51.4	
V45	116.8	178.9	66.2	31.1	22.5, 18.7		
Q46	120.5	175.1	57.2	31.7	26.3	180.8	Nε2: 113.5
D47[m]	118.5	173.3	52.3	38.4	175.1		
Q48	117.0	176.5	55.0	32.7	32.8	175.5	Nε2: 102.4

V49	122.7	173.9	62.3	34.6	22.4, 21.0		
L50	126.2	175.2	52.8	45.0	27.0	23.9	
L51	120.6	175.9	54.9	44.3	29.2	27.6, 26.2	
L52	124.0	176.9	53.2	44.9	27.6	23.7	
G53	119.4	174.7	47.2				
S54	122.7	173.0	58.1	63.7			
K55	123.0	175.4	55.3	33.8	24.4	29.3	Cε: 42.1
I56	126.3	176.7	62.5	37.6	29.4, 18.2	14.1	
L57	128.3	175.3	54.2	42.6	25.7	19.8	
K58	124.2	175.3	53.8	32.9	25.5	28.9	
P59	137.3	176.4	66.1	32.9	28.7	50.9	
R60[m]	110.3	177.7	56.8	29.7	25.3		
R61	120.9	175.9	56.2	31.7	28.9	43.2	
S62[m]	112.7	177.9	57.2	65.3			
L63	126.0	180.0	58.7	40.5	26.5	22.4, 14.4	
S64	113.0	177.8	61.0	62.6			
S65	119.4	175.0	61.0	62.8			
Y66[m]	119.8	176.1	59.4	37.4	132.5	132.6	Cε: 118.1, Cζ: 157.6
G67[m]	107.4		46.4				
D69[a]		173.6	58.0				
T73[a]	114.1	172.8	59.5	71.3	21.0		
H75	122.8	173.7	54.9	28.7	132.1	119.9	Cε1: 136.6, Nδ1:178.1, Nε2: 174.8
L76	122.0	176.8	52.6	45.2	27.1	24.7	
T77	119.0	172.4	61.6	71.0	22.0		
L78	126.8	175.2	53.4	44.6	27.8	23.6	
K79	128.8	174.0	54.8	34.4	25.1		
V80	122.7	176.5	61.2	33.7	22.0		
V81	128.0	174.1	62.4	32.3	21.6		

[a]Ambiguous. The backbone ^{15}N chemical shifts of K32 and K34 and their succeeding residues are nearly identical, making it difficult to tell them apart. The signals from L8, D69, and T73 are very weak. [m]For these residues, multiple conformations are observed.

7. Torsion angle analysis of microcrystalline N-FAT10-C0

Supporting information for Figure 2d (torsion angle plots)

- For residues P26-S29, D47, R61, and H75, there is no consensus among the TALOS-N database matches and, hence, no predicted torsion angles are shown.
- No angle predictions could be made for L8, I68-T73, and K82.
- Secondary structure predictions are based on observed chemical shifts except for G67, for which the prediction is based on sequence information.

Supporting information for Figure 2e (projection of secondary structure per residue)

- D69 and T73 are ambiguously assigned and colored yellow as secondary structure prediction is based on the sequence.
- Residues K32 and K34 are also ambiguously assigned but colored red (helix) as secondary structure prediction based on (very similar) chemical shifts is highly reliable.
- Residue I74 is unassigned but the secondary structure (light blue = strand) is still predicted based on chemical shifts of neighboring residues.

8. ^{13}C-^{13}C and ^{15}N-^{13}C spectra lyophilized/rehydrated N-FAT10-C0

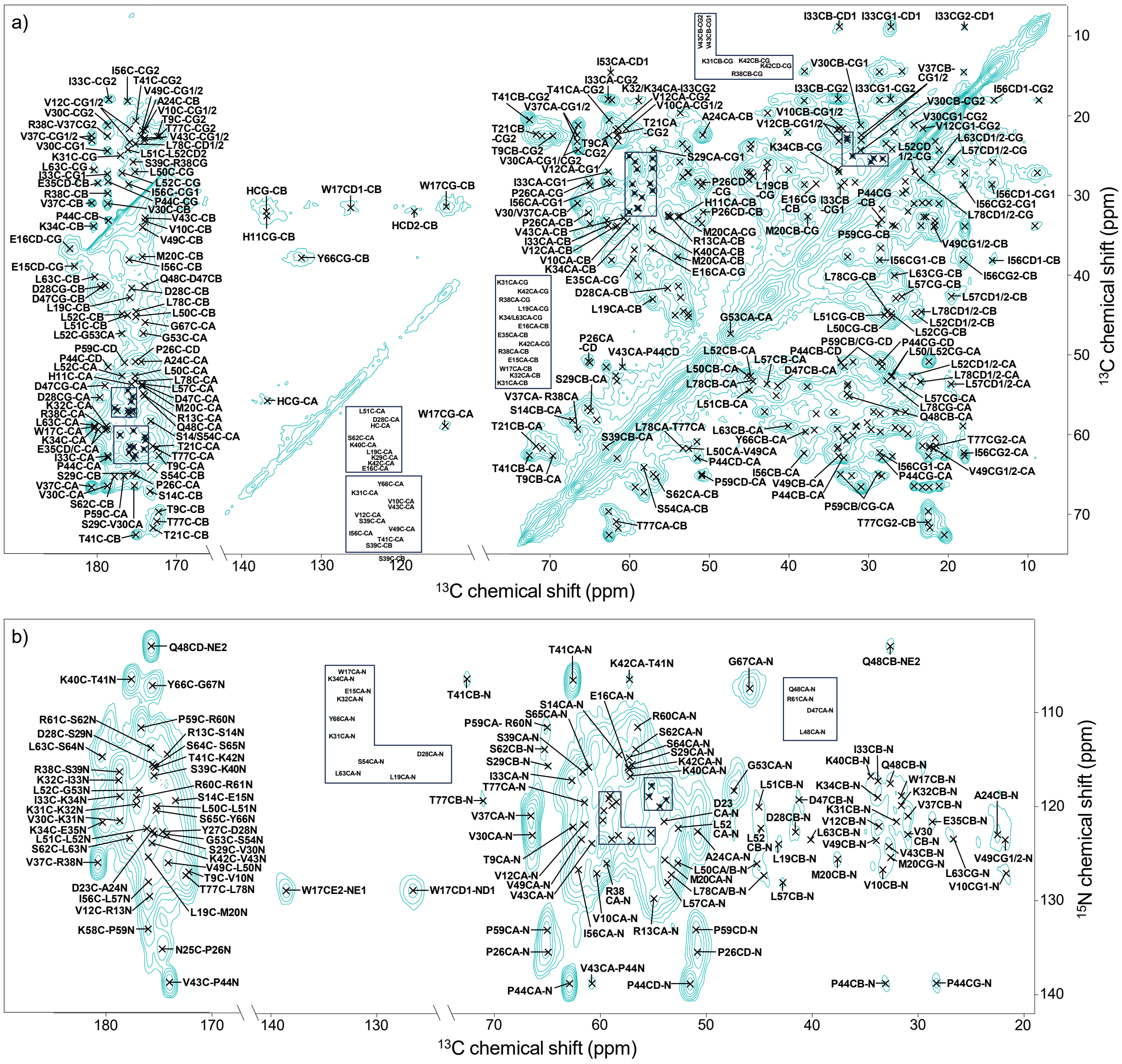

Figure S4: *(a) ^{13}C-^{13}C DARR and (b) ^{15}N-^{13}C ZF TEDOR spectra of lyophilized/rehydrated N-FAT10-C0. Sequentially assigned cross peaks are marked and labeled. As a result of the relatively long mixing time and the large number of scans for the given amount of protein, two- and three-bond N-C_β and N-C_γ correlations are visible in (b).*

9. Chemical shifts of lyophilized/rehydrated N-FAT10-C0

Table S4: *^{15}N and ^{13}C chemical shifts (ppm) of lyophilized/rehydrated U-^{13}C,^{15}N-N-FAT10-C0.*

Residue	N	C'	Cα	Cβ	Cγ	Cδ	Other
T9	122.2	172.2	62.6	69.6	22.5		
V10	127.0	174.0	60.3	33.2	21.6		
H11	125.4	175.7	53.7	32.6	137.0		
V12	121.9	175.9	61.5	33.7	23.2, 21.6		
R13	129.7	174.2	54.9	32.7			
S14	114.3	173.4	58.2	67.2			
E15	119.5	173.4	58.5	30.2	38.8	182.9	
E16	115.9	176.1	57.4	28.5	36.6	183.3	
W17	119.0	180.3	59.1	31.6	114.3	126.6, 124.7	Cε2: 138.5, Nε1: 128.8
L19	123.9	176.0	57.0	42.9	26.6		
M20	125.4	174.4	53.9	37.7	32.6		
T21	118.7	173.0	61.5	71.7	22.3		
D23	121.6	175.6	54.0	41.4	179.9		
A24	122.7	174.5	50.8	22.4			
N25[a]		174.6					
P26[a]	135.3	175.0	65.1	32.0	28.3	50.9	
Y27		174.7	54.4				
D28	122.7	175.6	55.2	41.6	179.7		
S29	115.7	175.4	57.0	64.9			
V30	123.0	178.7	66.4	31.0	24.3, 21.1		
K31	121.5	177.1	60.0	32.0	25.0		
K32	119.9	178.8	58.9	31.6	24.7		
I33	117.3	178.7	62.7	33.8	27.1, 18.0	8.8	
K34	118.9	180.3	59.2	33.9	26.6	28.4	
E35	121.7	179.0	59.4	28.5	36.8	179.9	
V37	120.9	180.7	66.5	30.9	23.2, 22.4		
R38	125.9	178.7	59.3	29.7	25.7		
S39	116.3	175.5	61.6	62.9			
K40	116.7	177.6	57.1	34.5			
T41	106.5	175.2	62.5	72.6	20.5		
K42	115.8	175.7	57.1	29.4	25.4	28.2	
V43	124.0	174.0	60.7	32.7	23.0, 22.8		
P44	138.9	178.6	62.9	33.1	28.3	51.5	
D47	119.3	174.2	53.8	41.3	179.0		
Q48	117.5	174.0	55.1	32.6	32.7	175.7	Nε2: 103.0
V49	123.5	174.2	61.8	33.9	21.8		
L50	126.1	175.2	52.6	45.2	27.1		
L51	120.0	176.1	54.4	45.0	28.0		
L52	122.6	176.9	52.7	44.9	27.2	24.2, 24.2	
G53	118.6	174.3	47.3				
S54	123.0	173.2	58.3	64.1			
I56	126.9	176.1	62.3	38.1	28.6, 18.1	14.5	
L57	128.1	174.4	53.7	42.7	25.8	19.6	
K58[a]		176.0	52.8				

P59[a]	133.2	176.6	65.0	33.5	28.8	51.0	
R60	111.6	177.1	56.5	29.8			
R61	119.1	175.8	55.4	32.0	28.0		
S62	113.9	177.8	56.6	65.3			
L63	123.5	180.4	58.9	40.1	26.7	22.1	
S64[a]	114.7	175.5	57.3	64.7			
S65	115.9	175.3	61.1	63.3			
Y66	120.5	175.5	59.7	37.9	132.6	133.6	Cε: 118.2, Cζ: 157.5
G67	107.1	173.8	45.9				
T77	119.3	172.5	61.7	71.0	22.5		
L78	127.3	175.2	53.3	44.6	27.7	23.5	
K79[a]	127.3		54.2	34.9			

[a]Ambiguous. Signals from P26 and P59 partially overlap and signals from their preceding residues (N25 and K58) are weak. The signals from S64 overlap with other serines. The signals from K79 are very weak.

10. Torsion angle analysis of lyophilized/rehydrated N-FAT10-C0

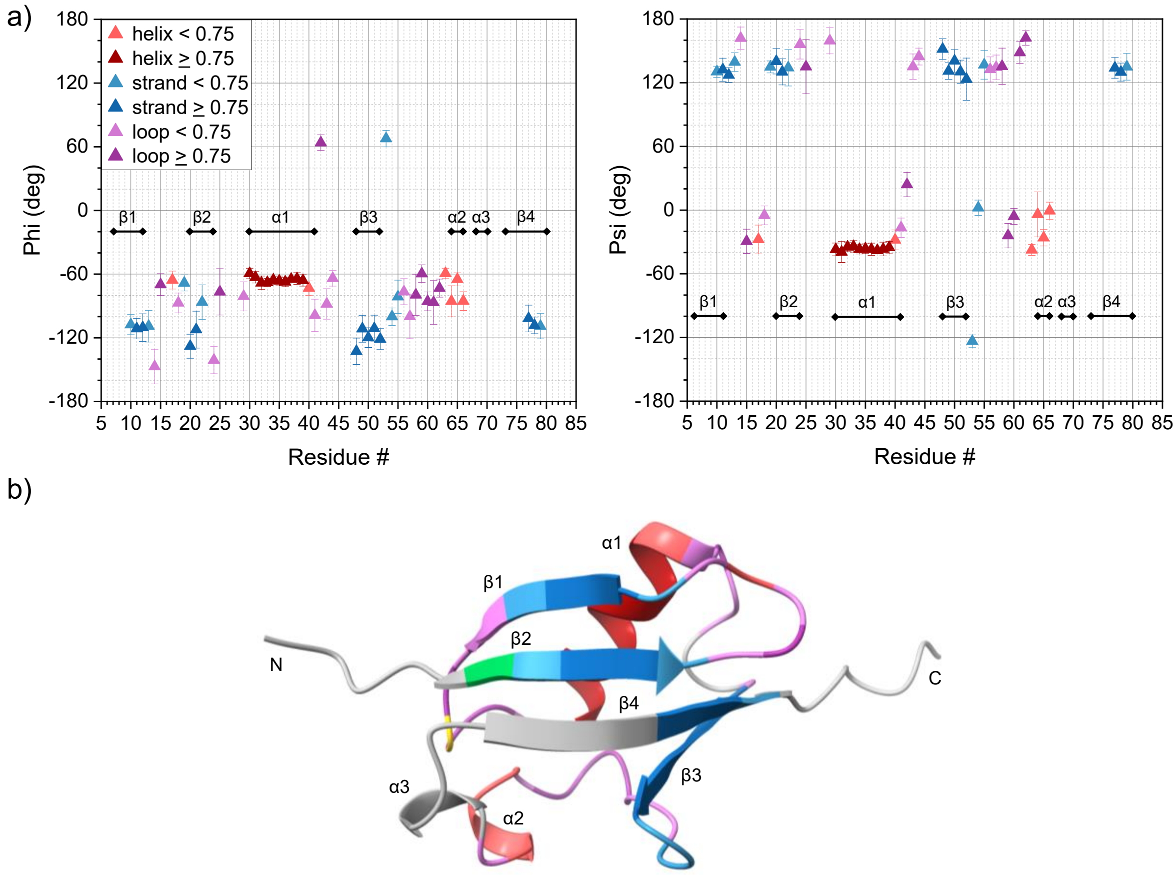

Figure S5: *(a) Backbone torsion angle and secondary structure predictions based on MAS NMR of lyophilized/rehydrated N-FAT10-C0. Error bars correspond to the standard deviation of the Φ and Ψ angles of the best matches in the TALOS-N database. For residues E16, D23, Y27, D28, D47, and G67 there is no consensus among the database matches and, hence, no predicted torsion angles are shown. No angle predictions are made for T9, P26, V45, Q46, I68-L76, and V80. Secondary structure predictions are based on observed chemical shifts and classified as helix, strand, or loop. Predictions with a confidence ≥ 0.75 are highly reliable. (b) Projection of TALOS-N secondary structure predictions per residue on the AlphaFold structure of N-FAT10-C0. D18, F22, H36 and K55 are unassigned, but secondary structure is predicted from the chemical shifts of neighboring residues. For residues N25, K58, P59, S64 and K79, the assignments are ambiguous, but since the secondary structure is reliably predicted from chemical shifts of also neighboring residues, they are color coded accordingly.*

11. ^{13}C-^{13}C and ^{15}N-^{13}C spectra of N-FAT10-C0 in complex with NUB1L

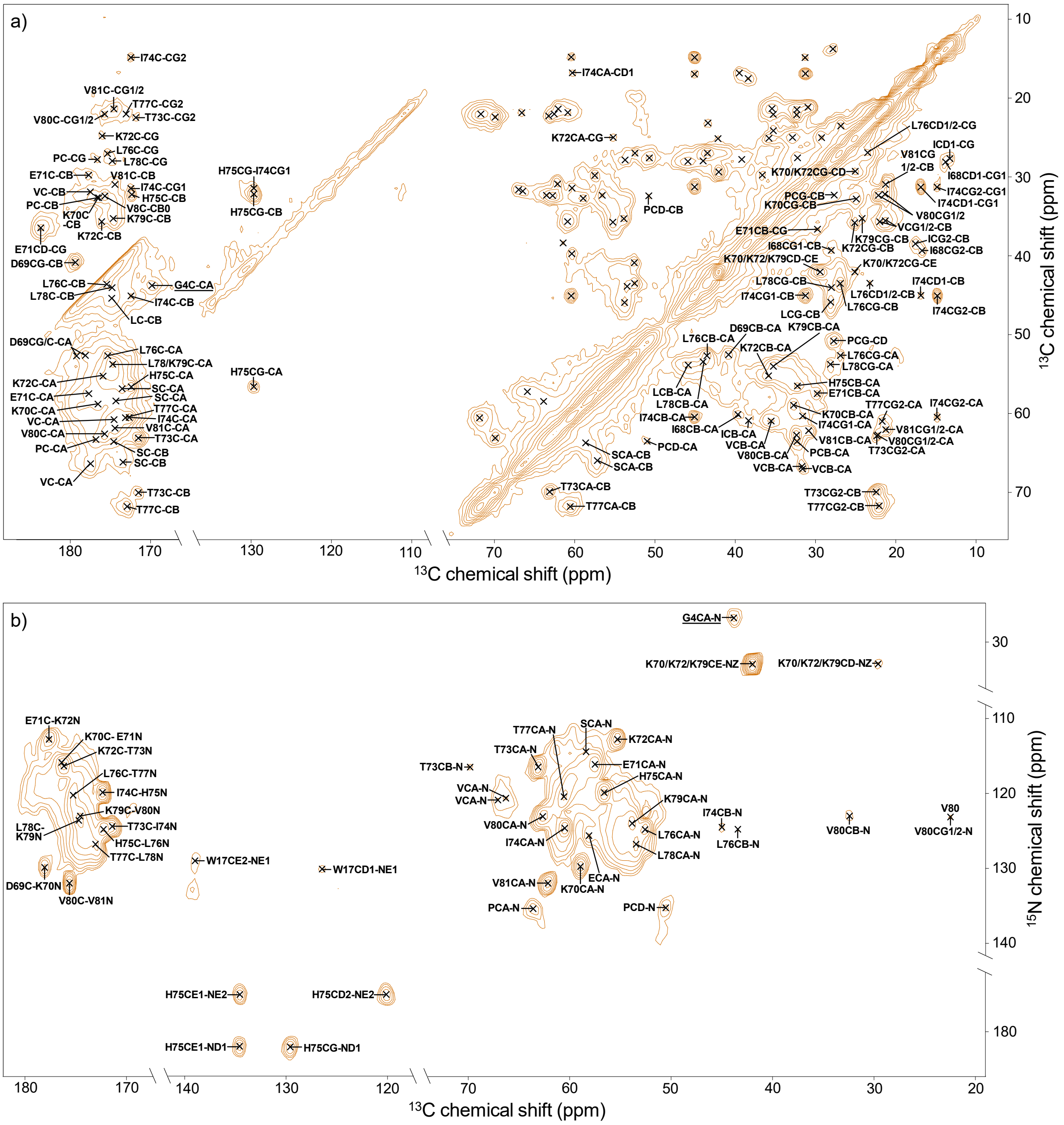

Figure S6: *(a) ^{13}C-^{13}C DARR and (b) ^{15}N-^{13}C ZF TEDOR spectra of N-FAT10-C0 co-sedimented with NUB1L. Sequentially assigned cross peaks are marked and labeled.*

12. Temperature dependence of N-FAT10-C0 in complex with NUB1L

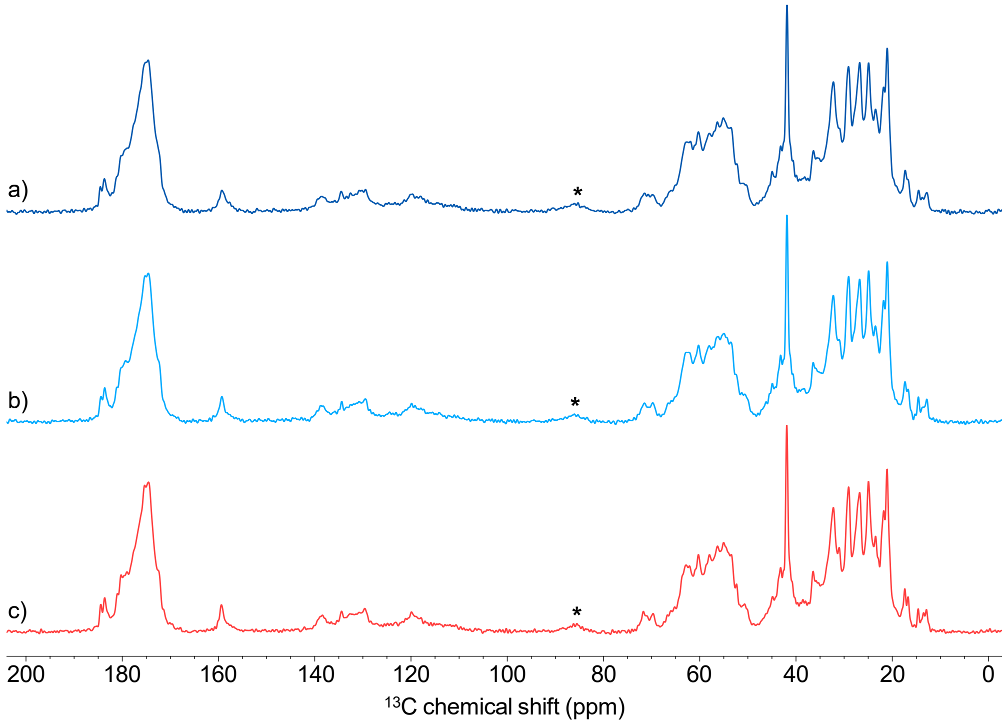

Figure S7: *^{13}C direct-excitation spectra of N-FAT10-C0 in complex with NUB1L at temperatures of (a) −10 °C, (b) −2 °C, and (c) 4 °C.*

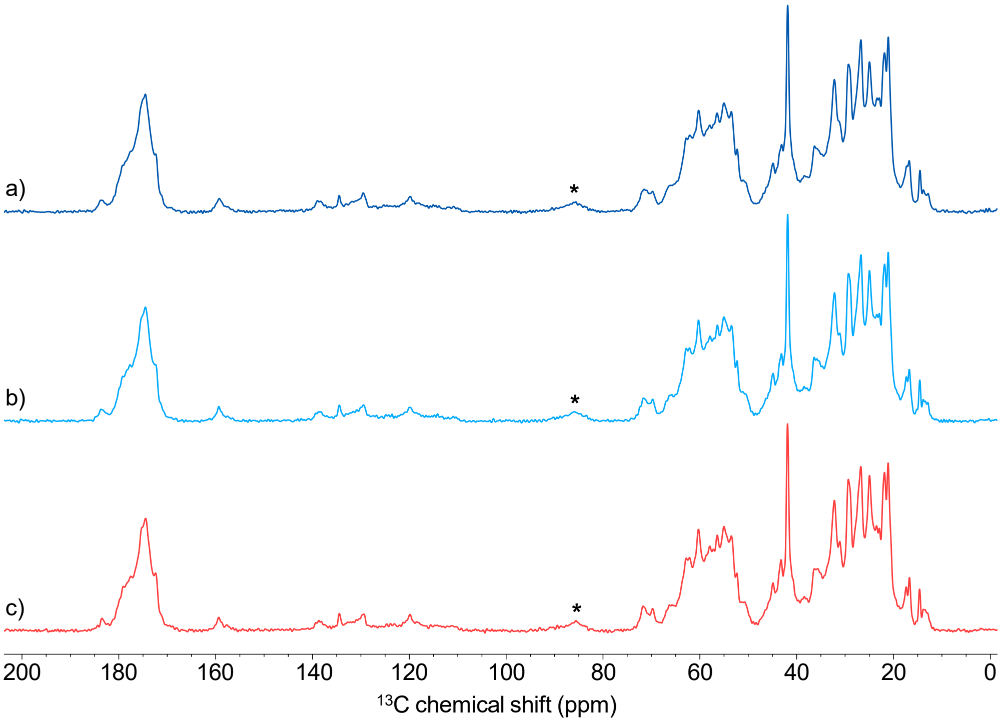

Figure S8: *^{1}H-^{13}C cross-polarization spectra of N-FAT10-C0 in complex with NUB1L at temperatures of (a) −10 °C, (b) −2 °C, and (c) 4 °C. Compared to the ^{13}C direct-excitation spectra, the signals in the carbonyl region are relatively weak. The reason is that the cross-polarization contact time of 1 ms does not allow a complete magnetization transfer to ^{13}C nuclei in this region, which, contrary to most other ^{13}C nuclei in a protein, do not have a directly bonded ^{1}H.*

13. Chemical shifts of N-FAT10-C0 in complex with NUB1L

Table S5: *^{15}N and ^{13}C chemical shifts (ppm) of U-^{13}C,^{15}N-N-FAT10-C0 co-sedimented with natural abundance NUB1L.*

Residue	N	C'	Cα	Cβ	Cγ	Cδ	Other
G4	26.8	169.6	43.8				
W17						126.3, 130.7	Cε2: 138.7, Cη2: 123.8, Cζ2: 114.4, Nε1: 129.6
I68			60.2	39.4	27.9, 16.8	13.6	
D69		178.1	52.6	40.8	179.2		
K70	129.6	176.5	58.9	32.7	25.0	29.3	Cε: 42.0, Nζ: 33.0
E71	115.9	177.6	57.5	29.7	36.6	183.6	
K72	112.7	176.1	55.3	35.8	25.0	29.3	Cε: 42.0, Nζ: 33.0
T73	116.4	171.5	63.1	70.0	22.4		
I74	124.4	172.5	60.4	45.1	31.3, 14.8	16.8	
H75	119.7	172.4	56.5	32.2	129.6	120.2	Cε1: 134.6, Nδ1: 182.1, Nε2: 175.4
L76	124.7	175.3	52.6	43.5	26.9	23.4, 22.1	
T77	120.3	172.9	60.6	71.8	22.0		
L78	126.8	174.7	53.6	44.0	27.9	22.1	
K79	123.6	174.6	53.7	35.2	24.3	29.4	Cε: 42.0, Nζ: 32.9
V80	123.0	175.6	62.5	32.3	22.1, 21.3		
V81	131.8	174.5	62.1	31.0	21.2		
E	125.6	181.2	58.1	31.2	36.6	184.3	
I			61.2	38.5	27.6, 17.5	13.1	
L		174.8	53.9	45.7	27.9		
P	135.1	176.7	63.5	32.4	27.7	50.7	
R						43.4	Cζ: 159.5, Nε: 85.2, Nη: 71.8
S	114.4	174.5	58.3	63.8			
S		173.5	57.0	66.0			
V	126.5	174.3	60.9	35.6	22.0, 21.3		
V	120.9		67.0	31.7			
V	120.6	177.4	66.5	31.8	21.7		
Y							Cζ: 157.7

14. Torsion angle analysis of N-FAT10-C0 in complex with NUB1L

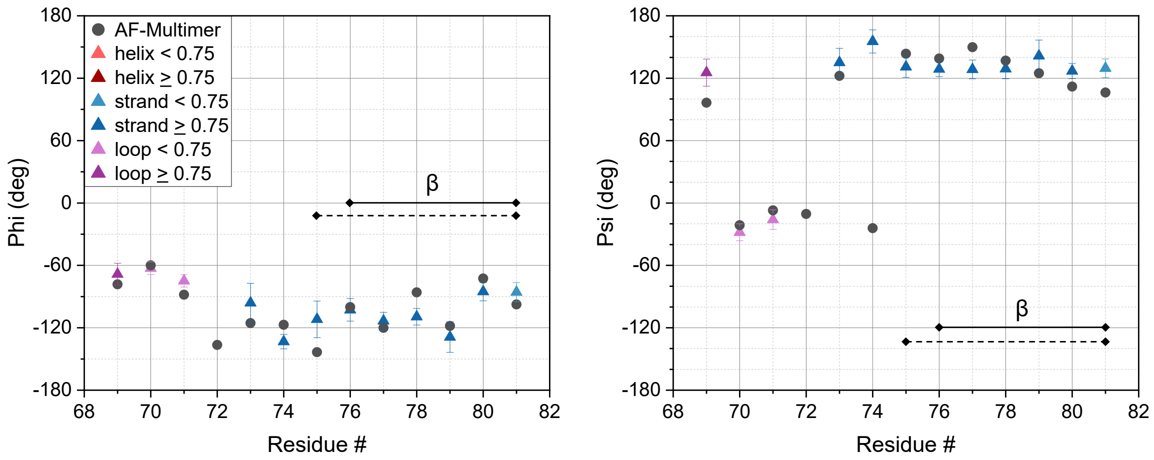

Figure S9: *Backbone torsion angle (triangles) and secondary structure predictions based on MAS NMR of N-FAT10-C0 co-sedimented with NUB1L. For comparison, torsion angles (black dots) of the AlphaFold-Multimer prediction (Figures 5a and S10) are also plotted for comparison. The horizontal solid and dashed black lines indicate the residues classified as β-strand by ChimeraX and VADAR, respectively. Error bars correspond to the standard deviations of the Φ and Ψ angles of the best matches in the TALOS-N database. For residue K72, there is no consensus among the database matches and, hence, no predicted torsion angles are shown. All plotted secondary structure predictions are based on observed chemical shifts. Based on sequence information, the secondary structure of I68 is classified as loop. Based on observed chemical shifts, the secondary structure of K72 is classified as loop. Predictions with a confidence ≥ 0.75 are highly reliable.*

15. AlphaFold-Multimer prediction for the interaction of N-FAT10-C0 and NUB1L

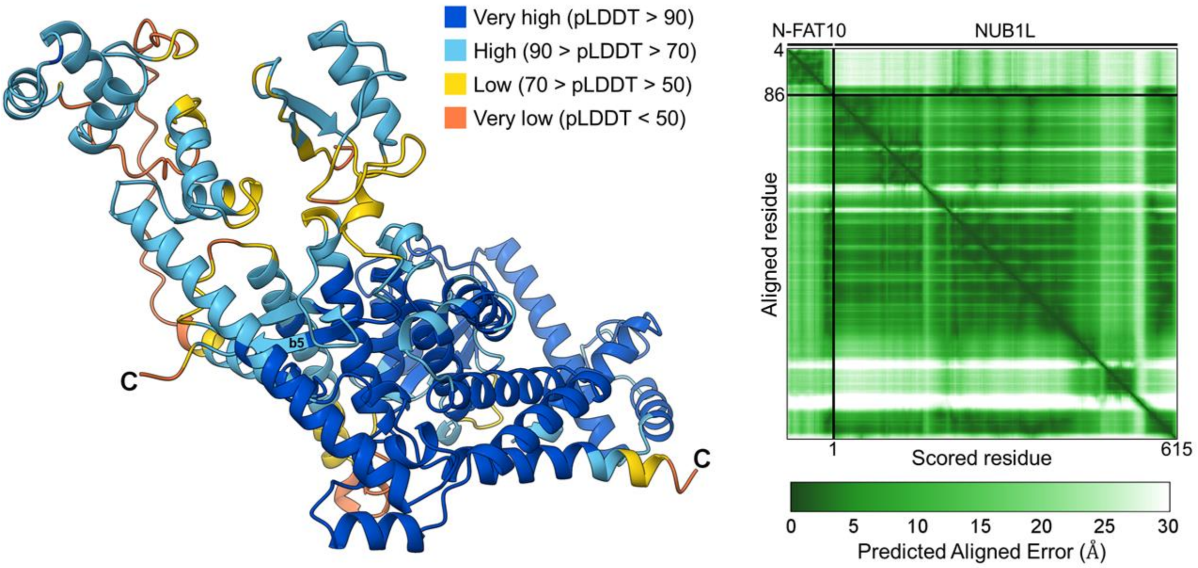

Figure S10: *(left) Best-ranked (0.8·ipTM + 0.2·pTM = 0.78) and relaxed AlphaFold-Multimer structure prediction for the interaction of N-FAT10-C0 and NUB1L. The color code indicates the pLDDT confidence score per residue. The predictions ranked 2-5 were similar, showing the intermolecular, anti-parallel β-sheet and with model confidence scores above 0.6. All other predictions scored poorly and showed no consistent interaction between N-FAT10-C0 and NUB1L. (right) Plot of the predicted aligned error (PAE). The small positional errors (< 5 Å, dark green) for residues I68 up to S84 of N-FAT10-C0 suggest that NUB1L tightly grasps the β5-strand.*

16. Double-REDOR experiments

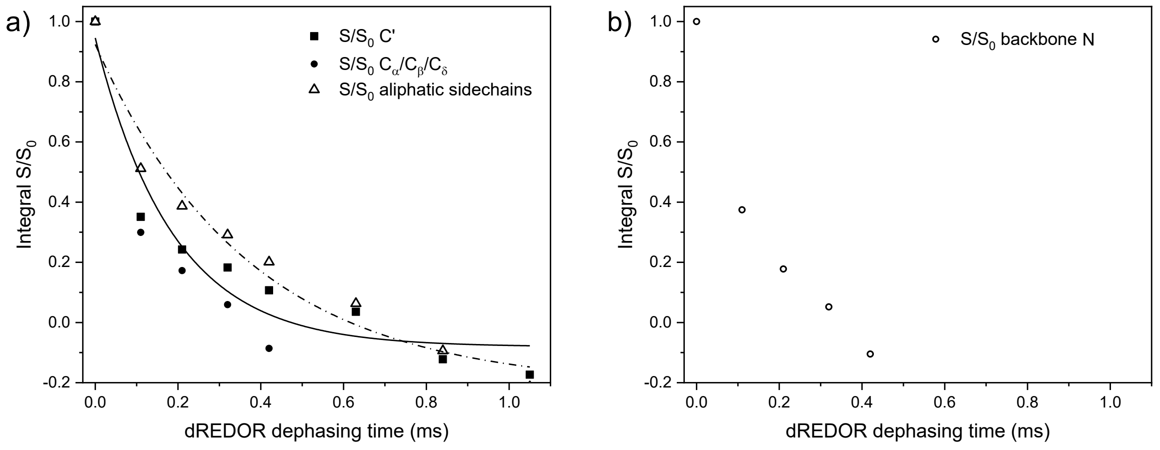

Figure S11: *Double-REDOR dephasing dynamics for (a) different types of* [13]*C nuclei and (b) backbone* [15]*N nuclei of isolated lyophilized/rehydrated N-FAT10-C0. Chemical shift ranges over which signal intensity with (*S*) and without (*S_0*) the double-REDOR dephasing pulses is compared, are indicated in Figures S12 and S13. Exponential decay functions (*$y = y_0 + Ae^{-x/\tau}$*, with* τ *the double-REDOR dephasing time) fitted to the experimental data points highlight that* ^{1}*H magnetization does not recover after dephasing for the carbonyl and aliphatic sidechain carbons. For the* C_α*,* C_β*,* C_δ *and the backbone* ^{15}*N nuclei, the transverse magnetization of the* ^{1}*H nuclei is lost too quickly after the initial 90°-pulse to properly capture the double-REDOR dephasing curve and it is not possible to fit an exponential decay.*

In their publication on its development, Polenova *et al.* note that the double-REDOR filter struggles to dephase the protons of the C_ε (42 ppm) of Lys.[13] We observe this behavior not only for this ^{13}C nucleus, but also for the N_ζ (33-34 ppm) of Lys and, in N-FAT10-C0 co-sedimented with NUB1L, the N_α of the N-terminus (26.8 ppm) and the $N_{\varepsilon 2}$ of His (175.4 ppm). We suspect that fast rotation of the ammonium group and/or ^{1}H chemical exchange are at fault. The dephasing behavior of the $N_{\delta 1}$ of His (182.1 ppm) could not be monitored, because the transverse ^{1}H magnetization is very quickly lost.

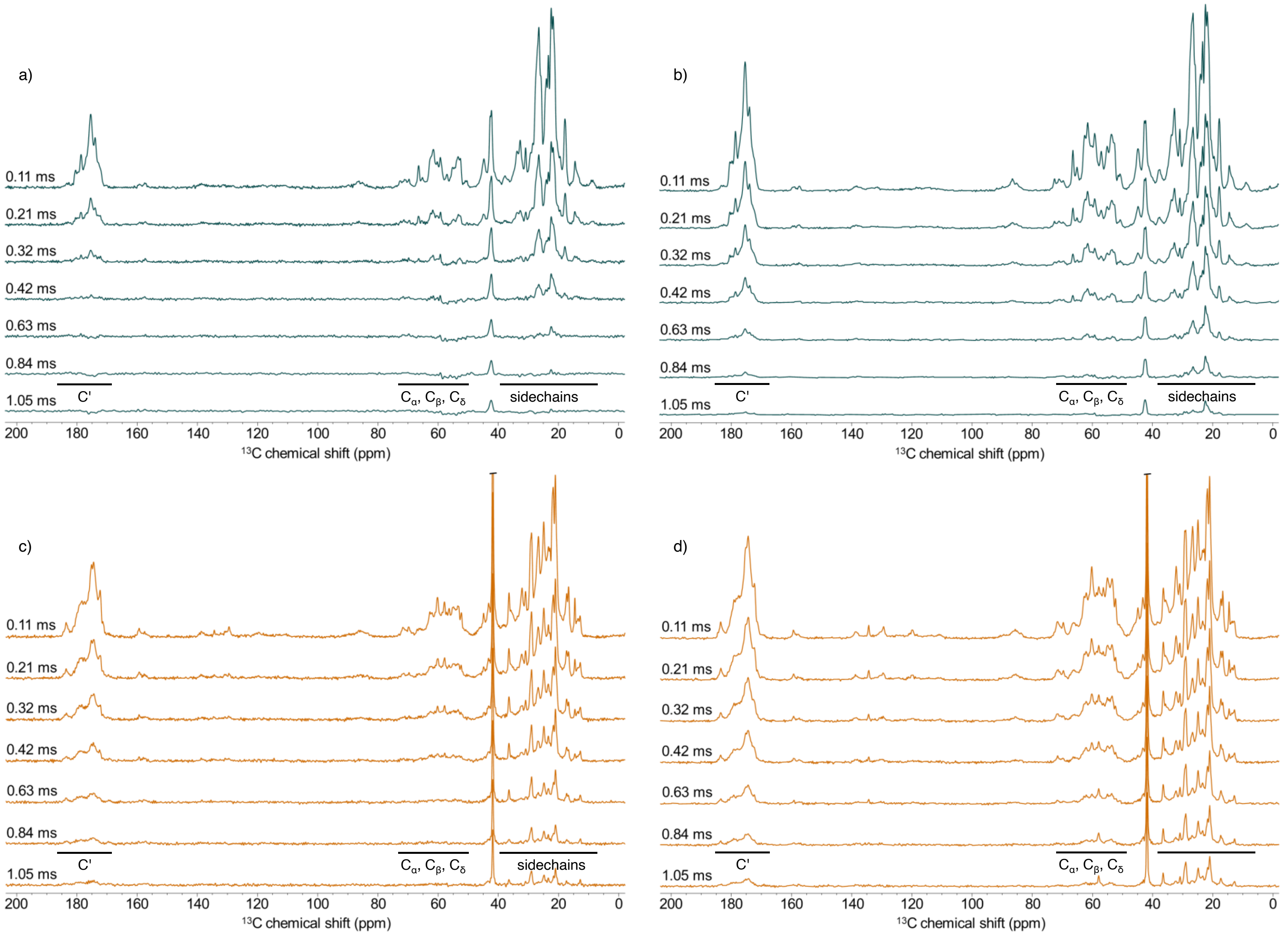

Figure S12: *^{1}H-^{13}C cross-polarization spectra of lyophilized/rehydrated N-FAT10-C0 (a,b) and of N-FAT10-C0 in complex with NUB1L (c,d) with the double-REDOR dephasing scheme inserted between the initial 90°-pulse and the contact period. The durations of the double-REDOR dephasing are indicated for each spectrum. In the spectra on the right (b,d), the power of the double-REDOR pulses is set to zero.*

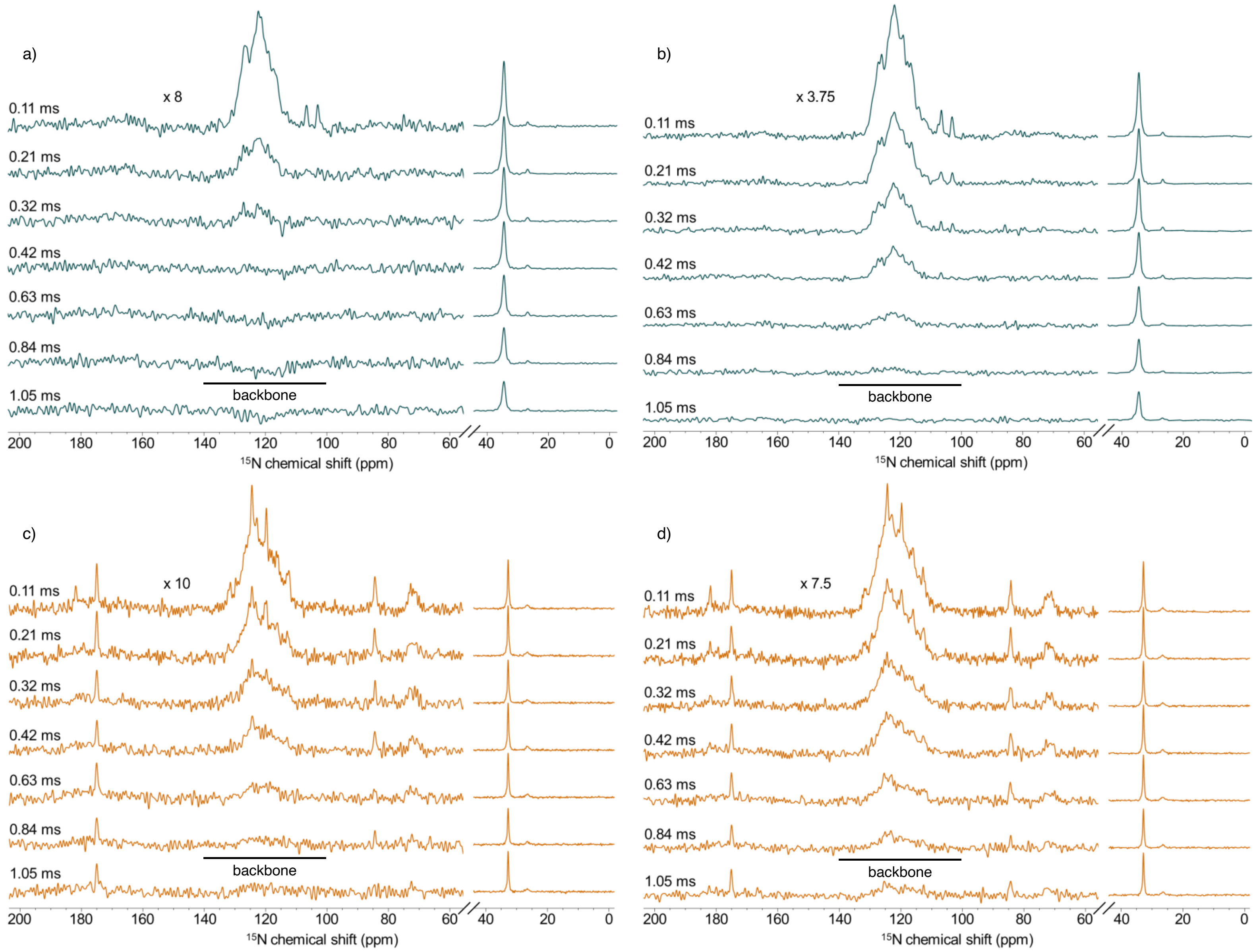

Figure S13: *^{1}H-^{15}N cross-polarization spectra of lyophilized/rehydrated N-FAT10-C0 (a,b) and of N-FAT10-C0 in complex with NUB1L (c,d) with the double-REDOR dephasing scheme inserted between the initial 90°-pulse and the contact period. The durations of the double-REDOR dephasing are indicated for each spectrum. In the spectra on the right (b,d), the power of the double-REDOR pulses is set to zero.*

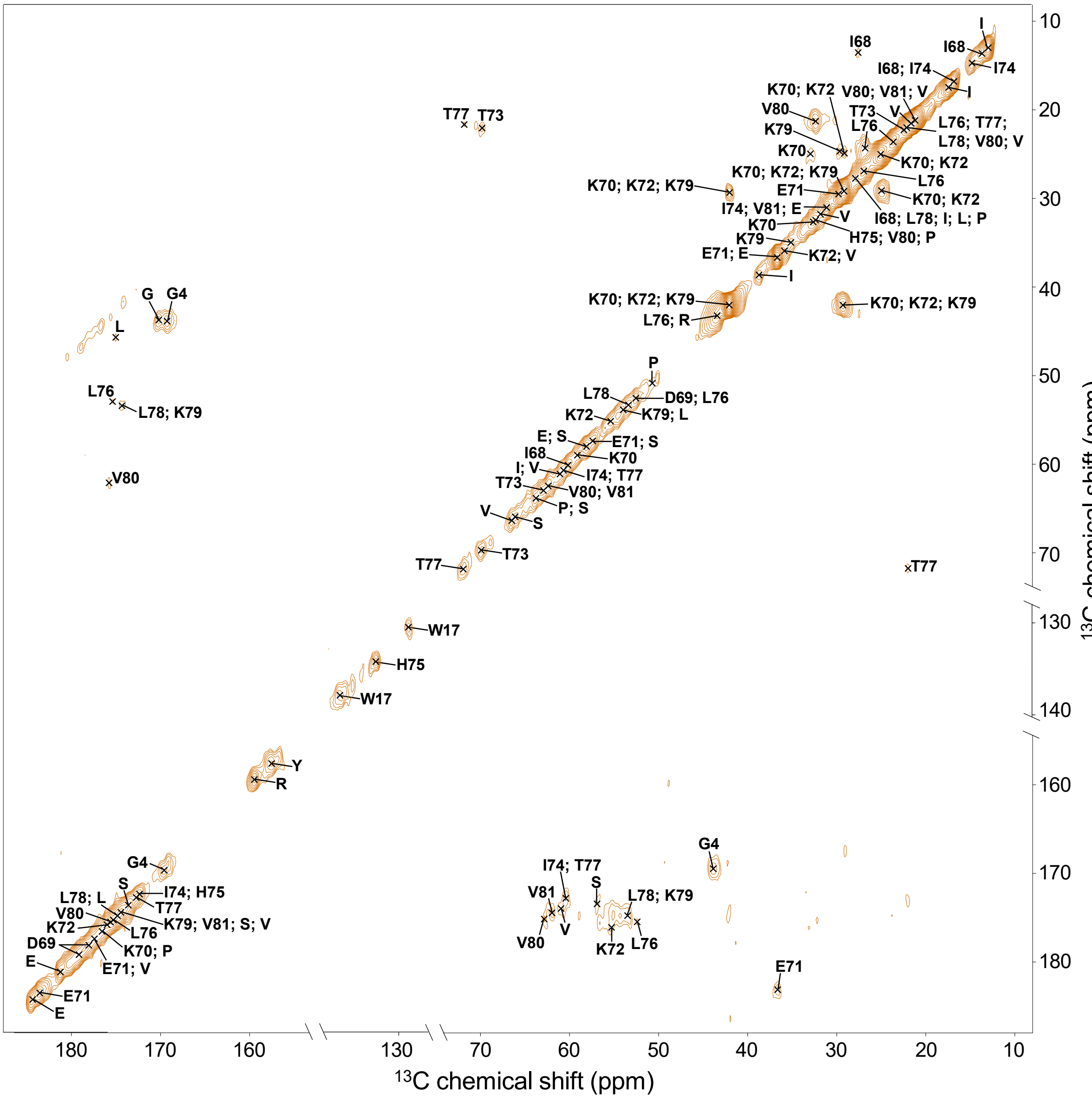

Figure S14: *^{13}C-^{13}C DARR spectrum of N-FAT10-C0 co-sedimented with NUB1L recorded after double-REDOR dephasing. All residues that are observed in regular ^{13}C-^{13}C and ^{15}N-^{13}C correlation spectra are observed in this spectrum as well, indicating close proximity to the non-dephased ^{1}H spins of NUB1L. The asymmetry of the cross-peak patterns across the diagonal is likely due to the weak replenishing of the proton magnetization in the C_α-region, i.e., magnetization is transferred from protons via C' to C_α nuclei, but much less via C_α to C' nuclei. The difficulty to dephase the N_α of the N-terminus is likely the reason that the C'-C_α cross peak of G4 at 169.6, 43.8 ppm shows up more strongly (and with a peak doubling of uncertain origin) than the C_α-C' cross peak at 43.8, 169.6 ppm on the other side of the diagonal.*

References

(1) Aichem, A.; Anders, S.; Catone, N.; Rößler, P.; Stotz, S.; Berg, A.; Schwab, R.; Scheuermann, S.; Bialas, J.; Schütz-Stoffregen, M. C.; Schmidtke, G.; Peter, C.; Groettrup, M.; Wiesner, S. The Structure of the Ubiquitin-like Modifier FAT10 Reveals an Alternative Targeting Mechanism for Proteasomal Degradation. *Nat. Commun.* **2018**, *9* (1), 3321. https://doi.org/10.1038/s41467-018-05776-3.

(2) Mandal, A.; Boatz, J. C.; Wheeler, T. B.; Van Der Wel, P. C. A. On the Use of Ultracentrifugal Devices for Routine Sample Preparation in Biomolecular Magic-Angle-Spinning NMR. *J. Biomol. NMR* **2017**, *67* (3), 165–178. https://doi.org/10.1007/s10858-017-0089-6.

(3) Thurber, K. R.; Tycko, R. Measurement of Sample Temperatures under Magic-Angle Spinning from the Chemical Shift and Spin-Lattice Relaxation Rate of 79Br in KBr Powder. *J. Magn. Reson.* **2009**, *196* (1), 84–87. https://doi.org/10.1016/j.jmr.2008.09.019.

(4) Morcombe, C. R.; Zilm, K. W. Chemical Shift Referencing in MAS Solid State NMR. *J. Magn. Reson.* **2003**, *162* (2), 479–486. https://doi.org/10.1016/S1090-7807(03)00082-X.

(5) Harris, R. K.; Becker, E. D.; Cabral De Menezes, S. M.; Granger, P.; Hoffman, R. E.; Zilm, K. W. Further Conventions for NMR Shielding and Chemical Shifts (IUPAC Recommendations 2008). *Pure Appl. Chem.* **2008**, *80* (1), 59–84. https://doi.org/10.1351/pac200880010059.

(6) Pines, A.; Gibby, M. G.; Waugh, J. S. Proton-enhanced NMR of Dilute Spins in Solids. *J. Chem. Phys.* **1973**, *59* (2), 569–590. https://doi.org/10.1063/1.1680061.

(7) Hartmann, S. R.; Hahn, E. L. Nuclear Double Resonance in the Rotating Frame. *Phys. Rev.* **1962**, *128* (5), 2042–2053. https://doi.org/10.1103/PhysRev.128.2042.

(8) Stejskal, E. O.; Schaefer, J.; Waugh, J. S. Magic-Angle Spinning and Polarization Transfer in Proton-Enhanced NMR. *J. Magn. Reson. 1969* **1977**, *28* (1), 105–112. https://doi.org/10.1016/0022-2364(77)90260-8.

(9) Thakur, R. S.; Kurur, N. D.; Madhu, P. K. Swept-Frequency Two-Pulse Phase Modulation for Heteronuclear Dipolar Decoupling in Solid-State NMR. *Chem. Phys. Lett.* **2006**, *426* (4), 459–463. https://doi.org/10.1016/j.cplett.2006.06.007.

(10) Fung, B. M.; Khitrin, A. K.; Ermolaev, K. An Improved Broadband Decoupling Sequence for Liquid Crystals and Solids. *J. Magn. Reson.* **2000**, *142* (1), 97–101. https://doi.org/10.1006/jmre.1999.1896.

(11) Marion, D.; Ikura, M.; Tschudin, R.; Bax, A. Rapid Recording of 2D NMR Spectra without Phase Cycling. Application to the Study of Hydrogen Exchange in Proteins. *J. Magn. Reson. 1969* **1989**, *85* (2), 393–399. https://doi.org/10.1016/0022-2364(89)90152-2.

(12) Andreas, L. B.; Eddy, M. T.; Chou, J. J.; Griffin, R. G. Magic-Angle-Spinning NMR of the Drug Resistant S31N M2 Proton Transporter from Influenza A. *J. Am. Chem. Soc.* **2012**, *134* (17), 7215–7218. https://doi.org/10.1021/ja3003606.

(13) Guo, C.; Hou, G.; Lu, X.; Polenova, T. Mapping Protein–Protein Interactions by Double-REDOR-Filtered Magic Angle Spinning NMR Spectroscopy. *J. Biomol. NMR* **2017**, *67* (2), 95–108. https://doi.org/10.1007/s10858-016-0086-1.
